# ^13^C-Urea Breath Test Accuracy for *Helicobacter pylori* Infection in the Asian Population: A Meta-Analysis

**DOI:** 10.5334/aogh.2570

**Published:** 2019-07-24

**Authors:** Muhammad Aklil Abd Rahim, Fadzrul Hafiz Johani, Shamsul Azhar Shah, Mohd Rohaizat Hassan, Mohd Rizal Abdul Manaf

**Affiliations:** 1 Department of Community Health, Faculty of Medicine, Universiti Kebangsaan Malaysia, Kuala Lumpur, MY; 2 Department of Community and Family Medicine, Faculty of Medicine and Health Sciences, Universiti Malaysia Sabah, Kota Kinabalu, Sabah, MY

## Abstract

**Background:**

*Helicobacter pylori (H. pylori)* infection is known to be associated with peptic ulcer and gastric cancer. Detection of H. pylori infection is a significant part of peptic ulcer and gastric cancer prevention and management. ^13^C-urea breath test (UBT) provides a good option for the pathogen detection due to its accuracy and safety.

**Objective:**

This review aims to evaluate the ^13^C-UBT diagnostic accuracy studies conducted among Asian population and validate its use for the Asian population.

**Methods:**

Original articles were systematically searched in PubMed, Scopus, and Google Scholar using the PICOS strategy by applying relevant keywords. Only studies published in English and conducted in Asia were included. Our search returned 276 articles. After assessment, 11 articles which answered our research question and met the criteria set for systematic review and meta-analysis were accepted. A total of 15 study protocols were extracted from the 11 accepted articles.

**Findings:**

Majority of the studies were conducted in Hong Kong (six), followed by Taiwan (five), Japan (two), and one each in Singapore and Israel. All studies had used histology as part of its gold standard of reference. All but one study was performed on adult populations. The summary estimate for sensitivity was 97% (95% CI: 96, 98%), and specificity was 96% (95% CI: 95, 97%), with significant heterogeneity between studies. Adjusting for the dose (50 mg) and breath sample collection time (20 minutes) had improved both accuracy estimates and significantly reduced heterogeneity.

**Conclusion:**

This review supports the test-and-treat strategy for *H. pylori* infection management. Prevalence and cost-effectiveness studies are mandatory for health authorities to adopt this strategy into national policy.

## Introduction

Gastric cancer remains a major public health concern, especially in Asia. It is one of the top five most prevalent cancers in the world, and a leading cause of mortality whereby it is one of the top three cancers with the highest number of annual mortality globally [1]. Gastric cancer incidence is the highest in East Asia, particularly Japan, South Korea and China [1]. Globally, *Helicobacter pylori* (*H. pylori*) is the major environmental cause of gastric cancer [1]. *H. pylori* infection has long been known to be associated with peptic ulcer and gastric cancer [2]. Detection of *H. pylori* infection is a significant part of gastric cancer prevention and management. ^13^C-UBT provides a good option for the pathogen detection due to its accuracy and safety [3]. However, differences between test kits may render the ^13^C-UBT tools from different manufacturers not suitable for the Asian population. The difference between test kits may be with regards to the dose of isotope, requirement to fast, timing of breath sample collection, usage or non-usage of a test drink to alter gastric emptying, and equipment for analysis [4]. There is also a major difference in terms of the carbon isotope used; either ^13^C or ^14^C. The ^13^C-UBT is preferred due to its non-radioactive nature and excellent sensitivity and specificity [5]. It is thus recommended by the Second Asia-Pacific Consensus Guidelines for *H. pylori* Infection that ^13^C-UBT tests are validated locally [4]. There have been a few meta-analyses to pool the sensitivity and specificity of ^13^C-UBT in diagnosing *H. pylori* infection, but no recent study had focussed on the Asian population. Those reviews had emphasized on different research questions; a review had assessed the test’s accuracy among children [6], another looked into population with partial gastrectomy done [7], and there was also a review in 2006 that concentrated on multiple *H. pylori* diagnostic tests (including ^13^C-UBT) in bleeding peptic ulcer patients [8]. Hence, this review aims to evaluate the ^13^C-UBT diagnostic accuracy studies conducted among Asian population and justify its use as a safe and accurate *H. pylori* detection tool validated for the Asian population.

## Materials and Methods

This review was conducted according to the Preferred Reporting Items for Systematic Reviews and Meta-Analyses (PRISMA) guidelines [9]. The study protocol was not registered.

### Search Protocol

A systematic search for articles published since inception until 2018 was conducted on PubMed, Scopus, and Google Scholar databases. Study titles, abstracts and keywords were searched by applying the PICOS strategy. Original articles were systematically searched using keywords of “Asia” for “P” (Population), “^13^C-Urea Breath Test” and its MeSH terms for “I” (Intervention), and “diagnostic accuracy”, “sensitivity”, “specificity”, and their MeSH terms for “O” (Outcome). There was no C (Comparison) or S (Study design) terms in the search protocol. Boolean operations namely “AND”, “OR”, or “NOT” were used to narrow and widen the search as according to the outlined objective. The initial studies obtained for first screening were retrieved from the final search algorithm of “P” AND “I” AND “O”. Only English language literatures and human studies were searched and included for this review. Other sources like unpublished reports, indirect article finding from bibliography of accepted articles, and grey literatures were not searched.

### Study Selection

Studies were first screened by the title. Studies with titles that do not conform to the objective of this review were immediately discarded. The remaining studies which were judged to be addressing the relevant research question were then randomly allocated to two reviewers for the screening of abstracts. As with the first stage of screening, studies deemed as not addressing the intended research question were excluded. Further assessment by two other reviewers followed after retrieval of the full text. Accepted studies were then subjected to systematic data extraction into a summary table with standardized headings. Studies were only included if: 1) it was conducted among Asian population; 2) it was an original article designed to measure the diagnostic accuracy of ^13^C-UBT for detection of *H. pylori* infection; and 3) the diagnostic accuracy metrics and measurements, namely true positive, true negative, false positive, and false negative numbers, were reported or were able to be indirectly extracted from the full-text. The exclusion criteria were: 1) the study had used ^13^C-UBT as the gold standard tool (either alone or in combination with other tools); 2) no English full text was available; and 3) inability to obtain the full text.

### Assessment of Study Quality

All accepted studies were assessed for quality using the Quality Assessment of Diagnostic Accuracy Studies (QUADAS-2) tool. This tool was validated for the purpose of assessing study quality of studies included in systematic reviews measuring diagnostic accuracy, and is an improvement of the original tool (QUADAS) published in 2003 [10,11]. The QUADAS-2 tool comprises of four key domains, namely patient selection, index test, reference standard, and flow and timing (of index test and reference standard). Although all domains were relevant for risk of bias assessment, only the former three domains were designed to assess applicability of each study to this review’s research question. There were no summary quality scores generated as it was deemed invalid for diagnostic accuracy systematic reviews [12]. QUADAS-2 was instead used to aid in selection of studies. Only studies which scored low risk of bias for reference standard and low risk of applicability concerns (all domains) were accepted. Studies were independently assessed by two reviewers and any discrepancy was resolved by consensus between the reviewers.

### Data Extraction

A standardized table with relevant headings was used to extract data. Extraction of studies included the identification of study locality, population characteristics (age group or other specific identifiers), name of the manufacturer-specific ^13^C-UBT used, characteristics of the ^13^C-UBT protocol used (dose of ^13^C-urea, presence/absence of test meal, duration from ingestion of carbon isotope to collection of post-ingestion exhaled air and others), the gold standard for *H. pylori* detection, total number of patients/respondents, and relevant diagnostic accuracy values. The extracted data regarding numbers of true positive, true negative, false positive, and false negative cases were also stated as to whether they were taken directly from the referenced text, or indirectly calculated or extrapolated from the available data. Studies were arranged in descending order according to year of publication, with the most recent study being placed in the first row.

### Statistical Analysis

Sensitivity, specificity, positive predictive value (PPV), and negative predictive value (NPV) were calculated for each study. Heterogeneity was assumed at significance level of p < 0.10 and was tested by chi squared. When heterogeneity was present, the degree was quantified using the I^2^ statistic. Values of less than 25% are considered as homogenous and 25% to <50% are considered as having low heterogeneity. For values of 50% or more, significant heterogeneity is assumed. Studies were then subjected to sub-group analyses to account for the heterogeneity between studies. Groups were not prespecified and were decided during analysis based on the criteria of having at least three studies in all sub-groups. All analyses were performed using the software Meta-Disc (version 1.4) [13].

## Results

### Study Selection

**Figure 1:**
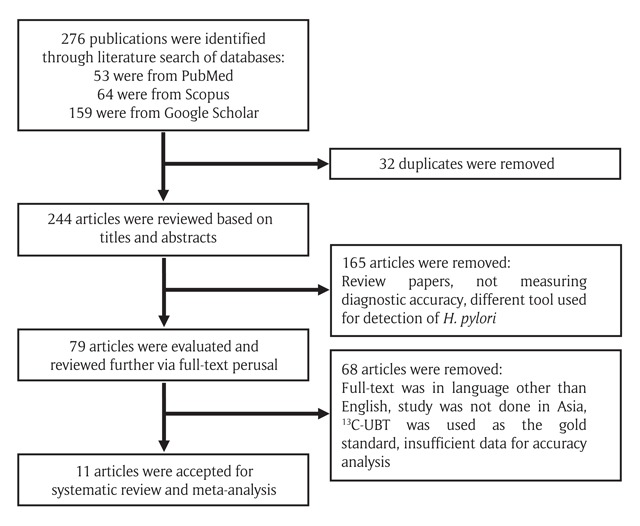
Flowchart of Study Selection for Meta-analysis.

The result of systematic search yielded 53 articles from PubMed, 64 articles from Scopus and 159 articles from Google Scholar, which totalled up to 276. After removal of duplicates (32), 165 articles were excluded after the screening of title and abstract, while a further 68 were omitted after full text review. The reasons for exclusion were due to full-text was in language other than English (2), study was not done in Asia (33), ^13^C-UBT was used as the gold standard or part of the gold standard (32), and insufficient data for accuracy analysis (1). The screening process resulted in 11 articles being accepted for systematic review and meta-analysis [14,15,16,17,18,19,20,21,22,23,24]. The flowchart of study selection is illustrated in Figure 1.

### Characteristics of Included Studies

Table 1 summarizes the quality assessment of the 11 accepted articles, along with the questions answered from QUADAS-2, while Tables 2 and 3 summarize the study characteristics of all included studies. Out of the 11 articles, there were considerable bias noted for six articles in the domain of index test [15,17,19,20,23,24]. This was due to the mentioned articles having index test protocol which had not prespecified the cut-off value for ^13^C-UBT used, which may result in an overestimation of the test performance. Two articles had unclear risk of bias for study selection [18,22]. In both articles, patient selection was based on an established condition or disease (partial gastrectomy patients and non-ulcer dyspepsia patients). This may lead to an exaggeration of diagnostic accuracy if the same test protocol was to be used on a different population [11]. However, all articles had low risk of bias for other domains, and there was no concern for applicability to the research question of this review.

**Table 1:** Summary of Quality Assessment of Diagnostic Accuracy Study (QUADAS-2) for Accepted Studies.

No.	First Author (Year)	Risk of Bias				Applicability Concerns		

		Patient Selection	Index Test	Reference Standard	Flow and Timing	Patient Selection	Index Test	Reference Standard

1.	Wardi (2012)	U	L	L	L	L	L	L
2.	Peng (2005)	L	H	L	L	L	L	L
3.	Urita (2004)	L	H	L	L	L	L	L
4.	Wong (2003)	L	L	L	L	L	L	L
5.	Chua (2002)	L	L	L	L	L	L	L
6.	Kato (2002)	L	H	L	L	L	L	L
7.	Wong WM (2001)	L	H	L	L	L	L	L
8.	Wong BCY (2001)	L	L	L	L	L	L	L
9.	Peng (2000)	U	L	L	L	L	L	L
10.	Wong (2000)	L	H	L	L	L	L	L
11.	Wang (1998)	L	H	L	L	L	L	L

L = Low risk. H = High risk. U = Unclear risk.

**Table 2:** Summary Table of Accepted Studies.

No.	First Author (Year)	Population & Country	Name of Test	Cut-off Threshold	Other Specific Features of Test	Gold Standard

1.	Wardi (2012)	Adults, partial gastrectomy, Israel	^13^C-BreathID	5.0 δ over baseline	75 mg, 4.5 g citric acid-based powder, breath sample at 10 to 15 minutes (delta time)	Histology
2.	Peng (2005a)	Adults, routine upper scope, Taiwan	^13^C-UBT (INER, Taiwan)	2.0 δ over baseline	50 mg, 6-hour fast, no test meal, breath sample at 15 minutes	Culture or histology + CLO
	Peng (2005b)	Adults, routine upper scope, Taiwan	^13^C-UBT (INER, Taiwan)	5.0 δ over baseline	100 mg, 6-hour fast, no test meal, breath sample at 15 minutes	Culture or histology + CLO
3.	Urita (2004)	Adults, diagnostic upper scope, Japan	Modified ^13^C-UBT (sample via nostril)	2.5 δ over baseline	100 mg, overnight fast, breath sample at 20 minutes	Histology + serology
4.	Wong (2003)	Adults, referred to upper scope unit, Hong Kong	Tablet ^13^C-UBT (Diabact UBT)	3.0 δ over baseline	50 mg, overnight fast, 456 mg anhydrous citric acid, breath sample at 20 minutes	RUT + histology
5.	Chua (2002)	Adults, referred to upper scope unit, Singapore	^13^C-UBT (Hp-Plus, Sweden)	3.5 δ over baseline	Amount of ^13^C-urea not stated, test meal with solution containing citric acid, breath sample at 30 minutes	Histology + CLO + serology (2/3)
6.	Kato (2002)	Children, referred to upper scope unit, Japan	^13^C-Urea Breath Test	3.5 δ over baseline	75 and 100 mg, 4-hour fast, no test meal, breath sample at 20 minutes	Culture or histology + RUT
7.	Wong WM (2001a)	Adults, referred to upper scope unit, Hong Kong	^13^C-Urea Breath Test	7.5 δ over baseline	50 mg, overnight fast, no test meal, breath sample at 20 minutes	Histology + CLO
	Wong WM (2001b)	Adults, referred to upper scope unit, Hong Kong	^13^C-Urea Breath Test	3.0 δ over baseline	50 mg, overnight fast, 2.4 g citrate solution as test meal, breath sample at 20 minutes	Histology + CLO
8.	Wong BCY (2001)	Adults, referred to upper scope unit, Hong Kong	^13^C-Urea Breath Test	5.0 δ over baseline	75 mg, overnight fasting, 2.4 g citric acid 200 mL test meal solution, breath sample at 30 minutes	Histology + RUT + CLO + culture + PCR
9.	Peng (2000)	Adults, non-ulcer dyspepsia, Taiwan	^13^C-UBT (INER, Taiwan)	3.0 δ over baseline	100 mg, 6-hour fast, 100 mL fresh milk as test meal, breath sample at 15 minutes	Culture or histology + CLO
10.	Wong (2000a)	Adults, referred to upper scope unit, Hong Kong	^13^C-Urea Breath Test	5.0 δ over baseline	75 mg, overnight fast, 2.4 g citric acid solution as test meal, breath sample at 30 minutes	Histology + CLO
	Wong (2000b)	Adults, referred to upper scope unit, Hong Kong	^13^C-Urea Breath Test	5.0 δ over baseline	75 mg, overnight fast, no test meal, breath sample at 30 minutes	Histology + CLO
11.	Wang (1998a)	Adults, routine upper scope, Taiwan	^13^C-UBT (INER, Taiwan)	3.0 δ over baseline	100 mg, 6-hour fast, 100 mL fresh milk as test meal, breath sample at 15 minutes	Histology or culture or urease test
	Wang (1998b)	Adults, routine upper scope, Taiwan	^13^C-UBT (INER, Taiwan)	4.0 δ over baseline	100 mg, 6-hour fast, 100 mL fresh milk as test meal, breath sample at 30 minutes	Histology or culture or urease test

PCR   Polymerase chain reaction. CLO   Campylobacter-like organism test (rapid urease test). RUT   Rapid urease test.

**Table 3:** Summary Table of Accepted Studies with Indicators of Diagnostic Accuracy.

No.	Author (Year)	Total, *n*	TP	TN	FP	FN	Sensitivity	Specificity	Overall Accuracy	PPV	NPV

1.	Wardi (2012)	76	^i^9	^i^57	^i^5	^i^5	64.2	91.9	86.8	64.2	91.9
2.	Peng (2005a)	50	27	22	0	1	96.4	100.0	98.0	100.0	95.6
	Peng (2005b)	50	18	32	0	0	100.0	100.0	100.0	100.0	100.0
3.	Urita (2004)	127	^i^42	^i^85	^i^0	^i^0	100.0	100.0	100.0	100.0	100.0
4.	Wong (2003)	200	^i^99	^i^101	^i^0	^i^0	100.0	100.0	100.0	100.0	100.0
5.	Chua (2002)	100	65	31	0	4	94.2	100.0	96.0	100.0	88.6
6.	Kato (2002)	220	^i^87	^i^129	^i^2	^i^2	97.8	98.5	98.2	97.8	98.5
7.	Wong WM (2001a)	101	^i^49	^i^50	^i^2	^i^0	100.0	96.2	98.0	96.1	100.0
	Wong WM (2001b)	105	^i^50	^i^54	^i^1	^i^0	100.0	98.2	99.1	98.0	100.0
8.	Wong BCY (2001)	294	^i^151	^i^127	^i^4	^i^12	92.6	96.9	94.5	97.4	91.2
9.	Peng (2000)	136	^i^76	^i^49	^i^6	^i^5	93.8	89.1	91.9	92.7	90.7
10.	Wong (2000a)	202	^i^110	^i^86	^i^2	^i^4	96.5	97.7	97.0	98.2	95.6
	Wong (2000b)	202	^i^108	^i^86	^i^2	^i^6	94.7	97.7	96.0	98.2	93.5
11.	Wang (1998a)	352	197	143	10	2	99.0	93.4	96.6	95.2	98.6
	Wang (1998b)	352	196	142	11	3	98.5	92.8	96.0	94.7	97.9

i    Figures indirectly derived from the original articles via deduction of other available figures. TP   True Positive. TN   True Negative. FP   False Positive. FN   False Negative. PPV  Positive Predictive Value. NPV  Negative Predictive Value.

Out of the included articles, four articles reported two protocol variations in the ^13^C-UBT accuracy evaluation. In these articles, each test protocol was counted as an individual study which is separate from the other. Thus, a total of 15 protocol comparisons (referred to as studies hereinafter) were included in the meta-analysis. Majority of the studies were conducted in Hong Kong (six), followed by Taiwan (five), Japan (two), and one each in Singapore and Israel. All studies had histology as its gold standard of reference or at least as part of the tools forming the gold standard. All but one study was performed on adult populations. The only study conducted among children was also the solitary study that had used two variations of the ^13^C-urea in the UBT (75 mg and 100 mg) for its subjects and reported the results jointly, owing to the different age groups [15]. Since results were pooled in that study, the different dosages of ^13^C-urea used were not reported separately in our meta-analysis.

### Overall Accuracy and Exploration of Heterogeneity

**Figure 2:**
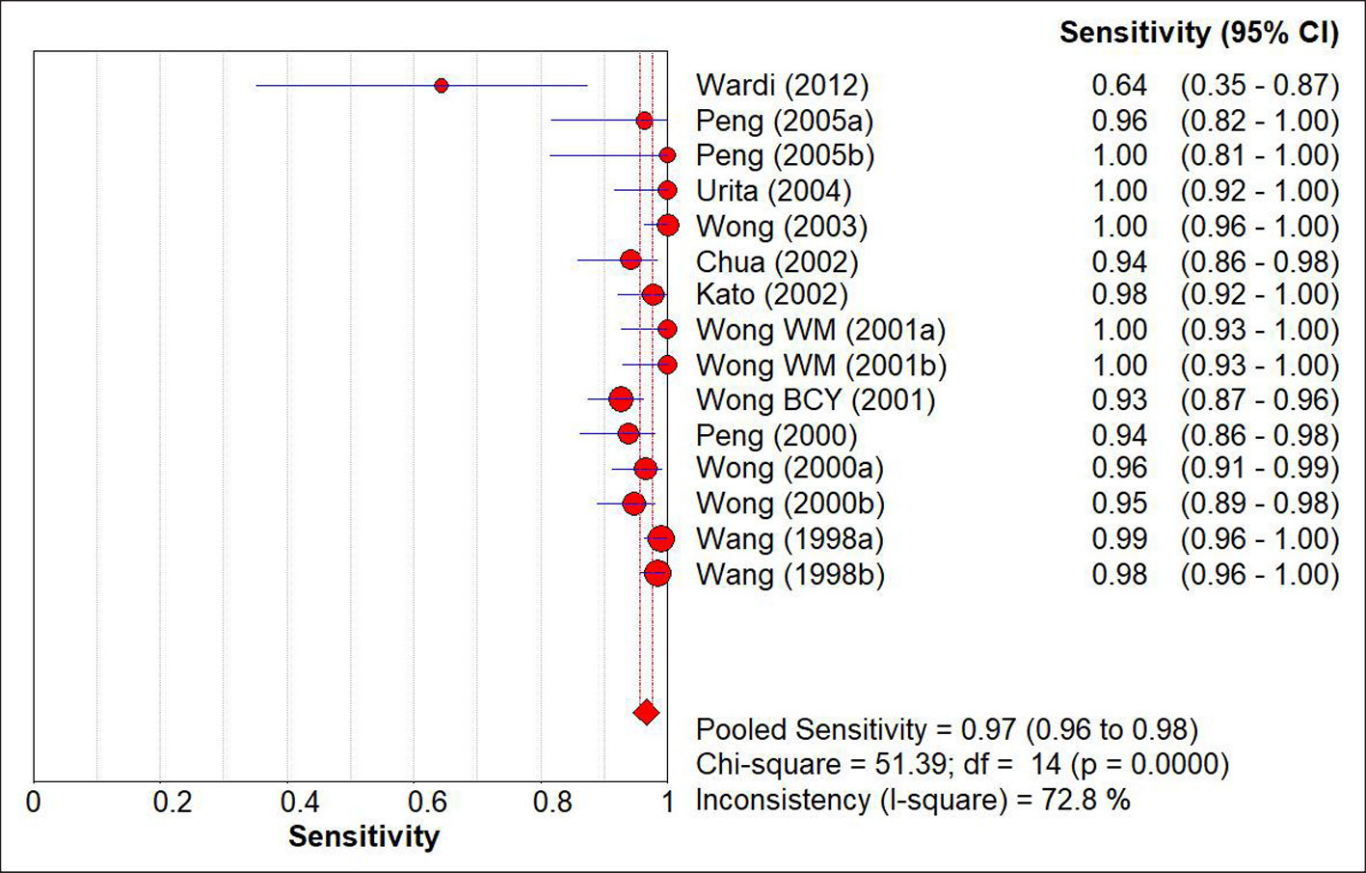
Overall Pooled Sensitivity of ^13^C-UBT to Detect *H. pylori* Infection.

**Figure 3:**
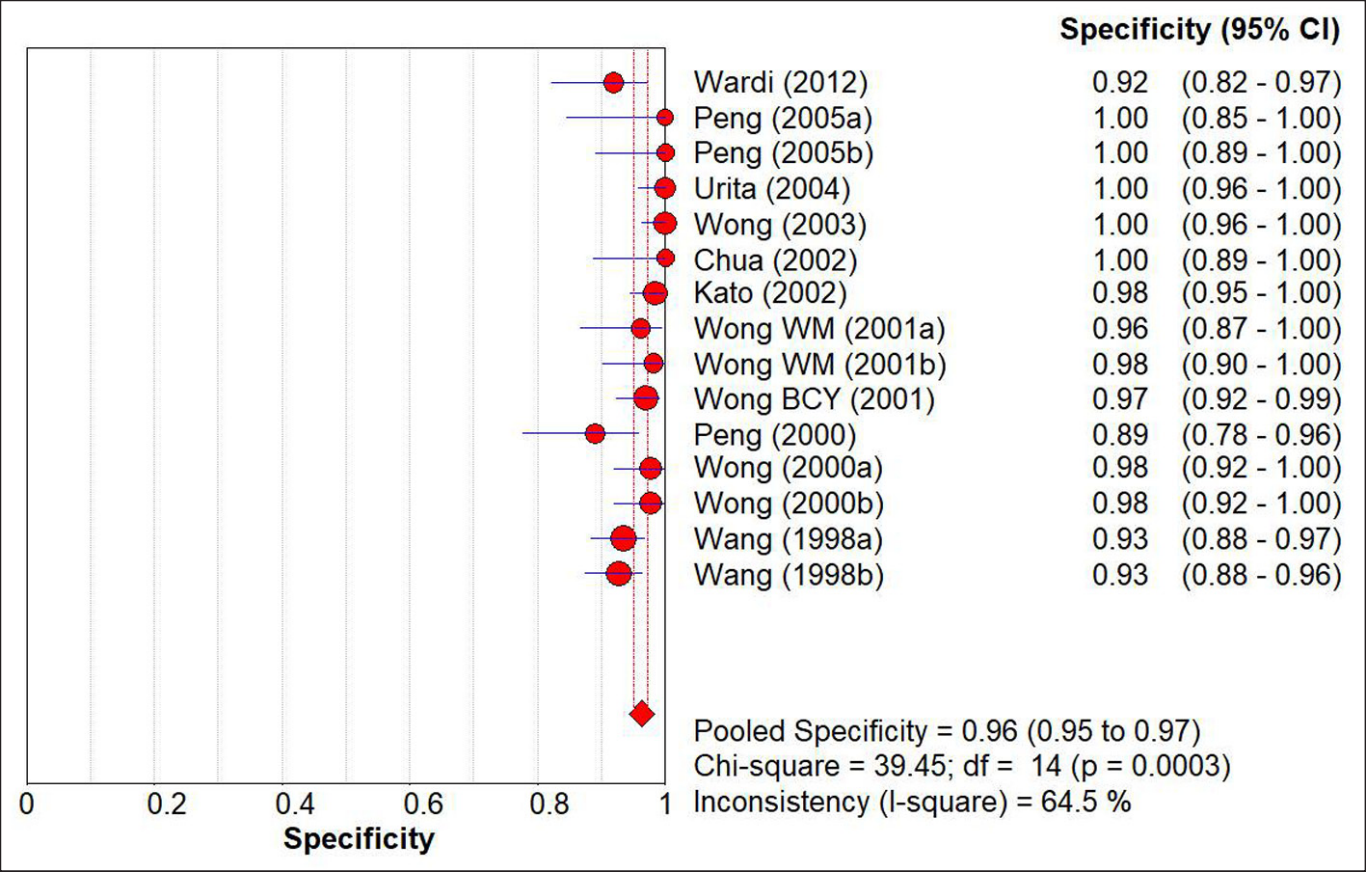
Overall Pooled Specificity of ^13^C-UBT to Detect *H. pylori* Infection.

When studies were pooled together, the summary estimate for sensitivity was 97% (95% CI: 96, 98%), and specificity was 96% (95% CI: 95, 97%) (Figures 2 and 3). While these were respectable numbers, suggesting a highly accurate test tool, the analysis was performed on studies which were largely heterogenous. Chi-squared p-values for both sensitivity and specificity pooled accuracy analyses were less than 0.1, indicating significant heterogeneity between the studies.

We conducted multiple sub-group analyses to stratify the studies into groups which were more homogenous. The summary statistics for the diagnostic accuracy of ^13^C-UBT to detect *H. pylori* infection and the findings of sub-group analysis were presented in Table 4. Dose of ^13^C-urea and breath sample collection time appeared to account for the largest variations in terms of heterogeneity of the outcome (pooled accuracy values). When adjusted according to the doses of ^13^C-containing urea used, studies with 50 mg in their ^13^C-UBT protocol had the best sensitivity (100%; 95% CI: 98, 100%) and specificity (99%; 95% CI: 96, 100%) compared to other protocols. The studies in this group were also homogenous (chi-squared p-values = 0.24 [sensitivity] & 0.17 [specificity]). Breath collection time showed similar accuracy improvement for protocols using 20 minutes as the time of breath sample collection following ingestion of ^13^C-urea. For these studies, the summary estimate for sensitivity was 99% (95% CI: 98, 100%) while for specificity it was similarly improved at 99% (95% CI: 97, 100%). These studies were also statistically homogenous. For other sub-groups, there was no apparent improvement of the diagnostic accuracy of the test after stratification, and heterogeneity persisted in at least one accuracy domain (sensitivity or specificity) analysis.

**Table 4:** Summary Statistics for the Diagnostic Accuracy of ^13^C-Urea Breath Test.

Sub-groups	Number of Studies	P-value (I^2^)_a_	P-value (I^2^)_b_	Sensitivity (95% CI)	Specificity (95% CI)

All studies*	15	<0.01 (72.8%)	<0.01 (64.5%)	0.97 (0.96, 0.98)	0.96 (0.95, 0.97)
By dose of ^13^C-urea**					
50 mg	4	0.24_#_ (28.7%)	0.17_#_ (40.6%)	1.00 (0.98, 1.00)	0.99 (0.96, 1.00)
75 mg	4	0.01 (76.5%)	0.28_#_ (21.0%)	0.93 (0.90, 0.96)	0.96 (0.94, 0.98)
100 mg	5	0.07 (53.0%)	<0.01 (76.1%)	0.98 (0.97, 0.99)	0.94 (0.92, 0.96)
**By cut-off threshold**					
<5.0 δ over baseline	9	0.02 (54.4%)	<0.01 (75.9%)	0.98 (0.97, 0.99)	0.96 (0.95, 0.97)
≥5.0 δ over baseline	6	<0.01 (76.5%)	0.30_#_ (17.3%)	0.94 (0.92, 0.96)	0.97 (0.95, 0.98)
**By breath sample collection time**					
10 to 15 minutes	5	<0.01 (83.0%)	0.06 (55.4%)	0.96 (0.94, 0.98)	0.94 (0.90, 0.96)
20 minutes	5	0.26_#_ (24.0%)	0.15_#_ (40.3%)	0.99 (0.98, 1.00)	0.99 (0.97, 1.00)
30 minutes	5	0.07 (54.6%)	0.10_#_ (48.4%)	0.96 (0.94, 0.97)	0.96 (0.94, 0.98)
By locality***					
Hong Kong	6	<0.01 (75.5%)	0.36_#_ (8.4%)	0.96 (0.94, 0.98)	0.98 (0.96, 0.99)
Taiwan	5	0.13_#_ (44.0%)	0.06 (54.8%)	0.98 (0.96, 0.99)	0.93 (0.91, 0.96)
**By manufacturer**					
^13^C-UBT (INER, Taiwan)	5	0.13_#_ (44.0%)	0.06 (54.8%)	0.98 (0.96, 0.99)	0.93 (0.91, 0.96)
Others	10	<0.01 (77.5%)	0.05 (46.7%)	0.96 (0.94, 0.97)	0.98 (0.97, 0.99)

* Four articles had data for two sets of samples, tools and findings; a total of 15 studies were obtained. ** One study was not included in this sub-group due to absence of reported ^13^C-urea amount used, and another study was excluded due to use of multiple amounts in a single study (for different age groups in children). *** Studies done in Japan, Israel and Singapore were excluded due to having only two or less studies per locality. ^a^ P-value for heterogeneity (chi-squared) and I^2^ test for heterogeneity quantification for sensitivity analysis. ^b^ P-value for heterogeneity (chi-squared) and I^2^ test for heterogeneity quantification for specificity analysis. _#_ P-value is not significant (≥0.1).

## Discussion

### Principal Findings

This systematic review had identified 15 study protocols from 11 articles that addressed the diagnostic accuracy of ^13^C-UBT to detect *H. pylori* infection in the Asian population. Our meta-analysis shows that: 1) ^13^C-UBT had outstanding diagnostic accuracy with sensitivity of 97% and specificity of 96%; 2) there were significant heterogeneity which persisted in most sub-group analyses performed; and 3) adjusting for the dose of ^13^C-urea (50 mg) and breath sample collection time (20 minutes) had improved accuracy estimates and significantly reduced heterogeneity, as to render the studies homogenous.

### Analysis of Heterogeneity

^13^C-UBT protocols differ between manufacturers, regions, and populations tested. There were multiple variables in a protocol for ^13^C-UBT procedure. These differences were likely to cause significant heterogeneity across the studies. Potential sources of heterogenous characteristics include the dose of ^13^C-urea used in test, cut-off threshold values, and breath sample collection time following ingestion of ^13^C-urea. The dose of ^13^C is usually determined by the manufacturer. For example, all studies that had used ^13^C-UBT manufactured by Institute of Nuclear Energy Research (INER), Taiwan, had 100 mg of ^13^C-urea in their ^13^C-UBT protocol, except for one study which had used both 50 mg and 100 mg variations of the test kit [18]. Cut-off threshold value represents a pivotal factor for diagnostic accuracy. The authors in six studies had not prespecified the cut-off values in their ^13^C-UBT protocols; instead using the ROC curve or manually calculating the best cut-off value from results to determine the best accuracy estimates via trade-offs between sensitivity and specificity [15,17,19,20,23,24]. A low cut-off threshold value may improve sensitivity but conversely reduce specificity, and vice versa. Regarding the breath sample collection time, it was also a matter of quid pro quo; too long and accuracy improves but may be inconvenient to patients, while too short and test is more convenient to both patient and operator, but may impair accuracy.

We attempted to diminish the effects of test protocol variations on heterogeneity by stratifying the studies according to the variations. For dose of ^13^C-urea used and breath sample collection time, our results showed that there was not only improvement in homogeneity of the studies, but also increased sensitivity and specificity (Table 4). However, it was only for ^13^C-urea dose of 50 mg and breath sample collection time of 20 minutes. For other sub-groups, significant heterogeneity persisted in analysis of at least one of the accuracy parameters (sensitivity or specificity). This was observed even for the five studies which had used the test from the same manufacturer (INER, Taiwan) [17,18,20]. This suggests that there were other possible sources of heterogeneity. Prior studies have argued that the test protocol itself is not solely responsible for the differences in test performance. Individual or patient characteristics also play a crucial role. Heterogeneity persisted in part because the ^13^CO_2_ exhaled (and collected in the sample collection bag) depends not only on the ^13^C-urea dose given and the amount hydrolysed by the urease from *H. pylori*, but also on individual attributes like the individual’s CO_2_ production, the degree of ^13^CO_2_ diluted within the body’s CO_2_ and bicarbonate pool, the anthropometric measurements, and important differentiating factors like age and sex [25,26]. We had stratified the studies into groups of similar localities in order to partly account for the differences between the populations tested, and it was noted that accuracy estimates did not improve, nor did the analysis for heterogeneity. The persistence of heterogenous qualities between the studies in this sub-group was expected, mostly due to our inability to further sub-divide the studies into groups which were more homogenous, in view of the limited number of studies.

### Epidemiologic and Policy Implications

Based on the results of our meta-analysis, ^13^C-UBT is an accurate tool to diagnose *H. pylori* infection, with the advantage of being non-invasive and safe. The use of ^13^C-UBT is recommended for the detection of *H. pylori* prior to eradication therapy, and post therapy to confirm eradication. The Second Asia-Pacific Consensus Guidelines for *H. pylori* recommended the test-and-treat approach, whereby uninvestigated dyspepsia patients with no alarm symptoms to suggest gastric cancer (such as dysphagia, weight loss, overt gastrointestinal bleeding, iron deficiency anaemia, or abdominal mass) are tested for *H. pylori* infection and managed accordingly [4]. This recommendation was echoed by the Maastricht V/Florence Consensus Conference [5]. It was argued that the test-and-treat approach, as compared to merely prescribing proton pump inhibitors (PPI) or directly performing oesophago-gastro-duodenoscopy (OGD), may provide better outcome, improved convenience for patients and greater cost-effectiveness in the long run. In an individual patient data meta-analysis conducted in 2005, comparing two arms between the test-and-treat and endoscope-and-treat patients, there was huge cost saving of US$389 per patient in the test-and-treat arm. However, the same meta-analysis also concluded that the endoscope-and-treat patients had a marginal but significant benefit in terms of symptom improvement and patient satisfaction [27].

The test-and-treat strategy must be appropriated with local settings. This approach is not recommended for regions with *H. pylori* infection prevalence of less than 10%, in which case the false positive rate may increase and causes unnecessary treatments [5]. Cost-effectiveness studies must also be conducted in similar settings prior to policy revamp. This is paramount in order to account for the local status regarding *H. pylori* prevalence, availability of resources and health services readiness. Further concern related to the test-and-treat strategy is regarding the delay of gastric cancer diagnosis. It was agreed that employing the test-and-treat strategy and possibly delaying referral for endoscopy for a brief time was unlikely to affect prognosis in gastric cancer [4]. A meta-analysis performed in 2009 on six randomised trials found that eradication of *H. pylori* reduces the risk of gastric cancer (relative risk: 0.65; 95% CI: 0.43, 0.98) [28]. In a more recent randomised placebo-controlled trial, compared to placebo, subjects who received *H. pylori* treatment had odds of 0.61 (95% CI: 0.38, 0.96) for gastric cancer incidence [29]. These evidences show that the eradication treatment will reduce the risk for gastric cancer and as such, an accurate and acceptable tool is mandatory for *H. pylori* detection. For the test-and-treat approach, ^13^C-UBT is an excellent tool to be used among the Asian population, due to its impeccable accuracy, convenience, non-invasiveness, and safety.

### Strengths and Weaknesses of Review

A major strength of this review is the study selection process, whereby two independent reviewers were involved in the screening of articles at all stages. There was also no limit placed for timeframe, resulting in reduced bias for study selection. We had also stratified the studies accordingly and attempted to explore the heterogeneity by adjusting for variations between the studies. This in turn had reduced the heterogeneity, most noticeably after stratifying the studies according to dose of ^13^C-urea in test protocol and the time of sample collection. Further, we had included all test protocols available in each article and analyse them separately during the process of meta-analyses, in order to improve homogeneity.

There were also weaknesses which are duly acknowledged. We had only included studies published in English and this may have introduced a measure of bias. Literatures in Japanese, Korean, and Chinese languages were not able to be included due to the authors’ lingual limitations. We also regret that during the process of exploring heterogeneity, a more comprehensive sub-group analysis was not able to be conducted due to the limited number of studies.

## Conclusion

To conclude, ^13^C-UBT is validated as an accurate tool for the diagnosis of *H. pylori* infection in the Asian population, with a sensitivity of 97% (95% CI: 96, 98%), and a specificity of 96% (95% CI: 95, 97%). Adjusting the test protocol for dose of ^13^C-urea and breath sample collection time may further improve accuracy estimates. The findings of this review support the test-and-treat strategy for *H. pylori* infection management and prevention of related diseases. Prevalence and cost-effectiveness studies are mandatory to aid health authorities to adopt this strategy into national policy. However, results must also be interpreted with considerations for the limitations, especially regarding the heterogenous nature of the test protocols included in this review.
